# The performance of a new rapid interferon gamma release assay based on fluorescence immunochromatography for *Mycobacterium tuberculosis* infection testing in village doctors in China

**DOI:** 10.1017/S0950268824001146

**Published:** 2024-11-08

**Authors:** Xuefang Cao, Yanxiao Chen, Henan Xin, Jiang Du, Boxuan Feng, Yijun He, Tonglei Guo, Lingyu Shen, Yuanzhi Di, Jianguo Liang, Zihan Li, Bin Zhang, Dakuan Wang, Zisen Liu, Weitao Duan, Qi Jin, Lei Gao

**Affiliations:** 1 NHC Key Laboratory of Systems Biology of Pathogens, National Institute of Pathogen Biology, and Center for Tuberculosis Research, Chinese Academy of Medical Sciences and Peking Union Medical College, Beijing, P. R. China; 2 Key Laboratory of Pathogen Infection Prevention and Control (Ministry of Education), National Institute of Pathogen Biology, Chinese Academy of Medical Sciences & Peking Union Medical College, Beijing, P. R. China; 3 College of Public Health; Zhengzhou University, Zhengzhou, China; 4 Hainan Medical University-The University of Hong Kong Joint Laboratory of Tropical Infectious Diseases, Key Laboratory of Tropical Translational Medicine of Ministry of Education, Hainan Medical University, Haikou, China; 5 The Center for Disease Prevention and Control of Zhongmu County, Zhengzhou, China

**Keywords:** AIMTB rapid test assay, interferon gamma release assay, *Mycobacterium tuberculosis*, village doctors, rapid diagnosis test

## Abstract

The AIMTB rapid test assay is an emerging test, which adopted a fluorescence immunochromatographic assay to measure interferon-γ (IFN-γ) production following stimulation of effector memory T cells in whole blood by mycobacterial proteins. The aim of this article was to explore the ability of AIMTB rapid test assay in detecting *Mycobacterium tuberculosis* (MTB) infection compared with the widely applied QuantiFERON-TB Gold Plus (QFT-Plus) test among rural doctors in China. In total, 511 participants were included in the survey. The concordance between the QFT-Plus test and the AIMTB rapid test assay was 94.47% with a Cohen’s kappa coefficient (κ) of 0.84 (95% CI, 0.79–0.90). Improved concordance between the two tests was observed in males and in participants with 26 or more years of service as rural doctors. The quantitative values of the QFT-Plus test was higher in individuals with a result of QFT-Plus-/AIMTB+ as compared to those with a result of QFT-Plus-/AIMTB- (*p* < 0.001). Overall, our study found that there was an excellent consistency between the AIMTB rapid test assay and the QFT-Plus test in a Chinese population. As the AIMTB rapid test assay is fast and easy to operate, it has the potential to improve latent tuberculosis infection testing and treatment at the community level in resource-limited settings.

## Background

Tuberculosis (TB) is a global public health problem, with nearly 10.6 million people developing TB disease and 1.3 million deaths worldwide in 2022 [1]. It is reported that approximately 25% of the global people is infected with *Mycobacterium tuberculosis* (MTB) [1,2], of which 5%–10% may progress to active disease during their lifetime [3]. To effectively reduce TB incidence, the detection and treatment of latent tuberculosis infection (LTBI) should be promoted to protect high-risk populations from developing active disease [1]. Currently, MTB infection test lacks gold standard, and immunological tests based on specific cellular immune responses are widely used [4,5]. The tuberculin skin test (TST) is an in vivo experimental method, which can be performed outside a laboratory. The limitations of it include that some proteins of tuberculin are also present in Bacille Calmette-Guérin (BCG) vaccination and nontuberculous mycobacterial (NTM), and it needs a return visit to measure the result [6]. Interferon gamma release assays (IGRAs) are in vitro immune detection methods that mainly detect the production of interferon-γ (IFN-γ) after MTB-specific antigen stimulation. Such in vitro testing, as compared with TST, greatly avoids false positivity caused by NTM infection or prior BCG vaccination [7]. However, IGRAs require extensive laboratory infrastructure and well-trained technicians. Hence, to further improve detection throughput and facilitate use in resource-limited settings, IGRAs based on other detection technologies have also become a hot topic for development.

The AIMTB rapid test assay (Leide Biosciences Co., Ltd., Guangdong, China) is a newly developed domestic test based on the IGRA principle and adopted a fluorescence immunochromatographic assay to detect a method for stimulating T cells to secrete IFN-γ by specific antigens early secreted antigenic target 6 [ESAT-6] and culture filtrate protein [CFP-10]. Its outstanding advantage is that the tedious post-stimulation steps are eliminated, and the detection can be completed in about 20 min for each sample. Another advantage is that its price would be lower than currently available testing products, making it more suitable for LTBI screening at the community level in areas with limited resources. However, the AIMTB rapid test assay has not been approved in China, and its performance needs to be systematically evaluated in varied populations. Village doctors are considered high-risk groups for MTB infection as they extensively participate in TB control, especially in patient management. Therefore, this article aimed to assess the performance of AIMTB rapid test assay in detecting MTB infection in rural doctors.

## Materials and methods

### Study design and population

Between August 24 and August 26, 2023, we undertook a cross-sectional study among registered rural doctors from Zhongmu County, Henan Province to assess the performance of AIMTB rapid test assay using the QuantiFERON-TB Gold Plus (QFT-Plus) test (Qiagen; Valencia, CA, USA) as reference. The study population would be included with the following criteria: having voluntarily participated in this study; being licensed physician; willing to participate in all research content. The study population would be excluded with the following criteria: current active TB or ever been diagnosed with TB; pregnant, women who were preparing to become pregnant or lactating women.

### Data collection

Trained interviewers collected the sociodemographic information of each participant through standardized questionnaires, including age, sex, educational level, income per month, smoking status, alcohol drinking status, weight, and height. In addition, information on MTB exposures was collected as well, including years of service as a rural doctor, whether to manage patients with TB in the past 4 years, and history of TB. Each participant was questioned about suspected symptoms of pulmonary TB and underwent a digital radiography examination to identify suspected active patients with TB. All suspected cases were transferred to TB-designated medical institutions for confirmation.

### Blood collection and laboratory procedures

Blood samples for each participant were collected in lithium heparin tubes for the QFT-Plus test and the AIMTB rapid test assay. Both tests were conducted strictly according to the manufacturer’s recommendations.

For the QFT-Plus test, 1 mL of whole blood was transferred to each tube (nil, TB1, TB2, and mitogen). Shaked and incubated for 20 h (37°C). After incubation, the samples were centrifuged for 15 min (3,000 × relative centrifugal force [RCF] (g)). Then, the released IFN-γ was tested by ELISA. The definition of QFT positive: the IFN-γ values in TB1-Nil or TB2-Nil ≥0.35 IU/mL and ≥ 25% of the Nil value, while the negative result was reversed. The definition of QFT is indeterminate: the IFN-γ value in the Nil tube was greater than 8.0 IU/mL or the IFN-γ value in Mitogen-Nil was less than 0.5 IU/mL.

**Figure 1.**
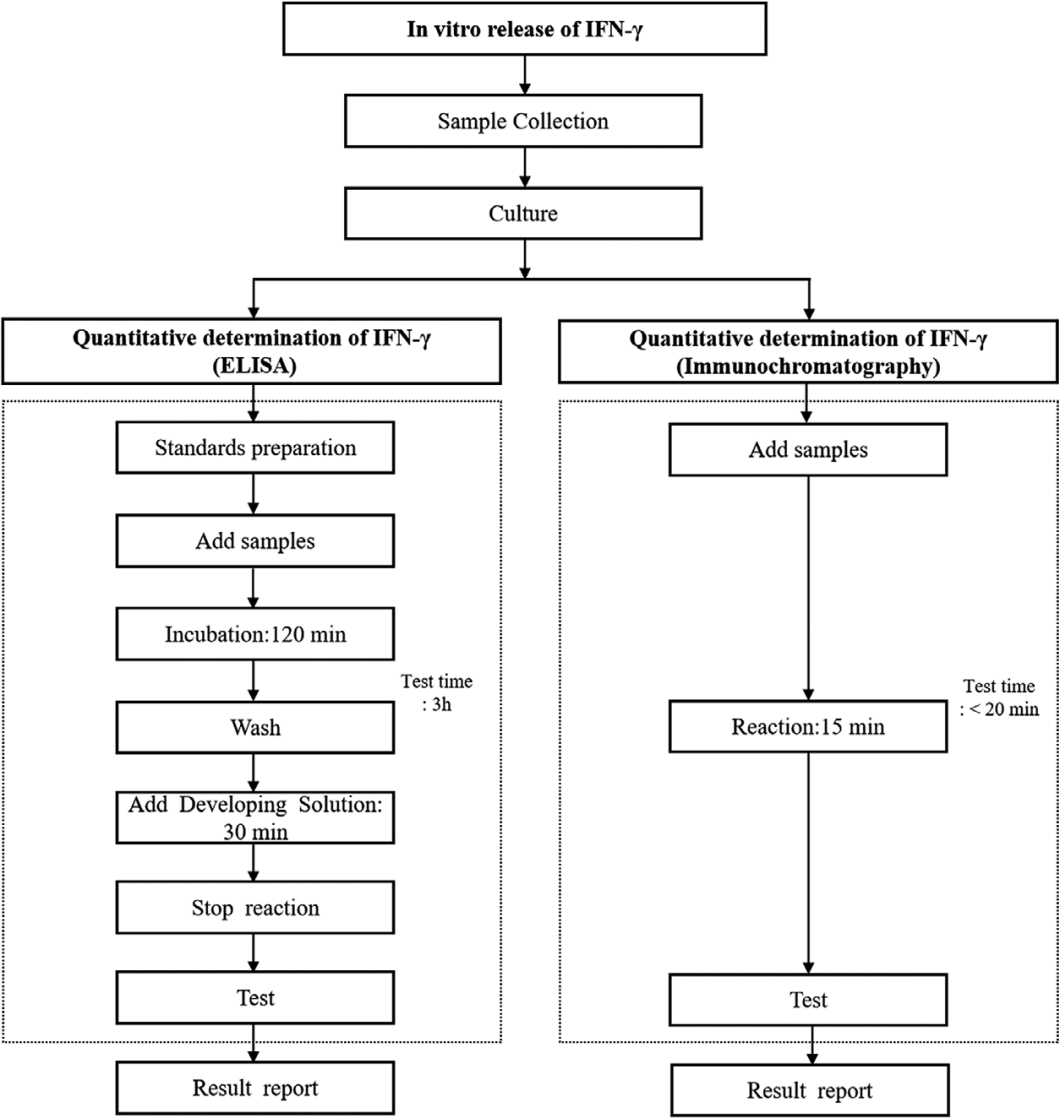
Operation procedure of enzyme-linked immunosorbent assay and immunochromatography.

For the AIMTB rapid test assay, 0.6 ml of whole blood was collected into each tube: N (negative control), T (TB antigen), and P (positive control). Shaked and incubated at 37°C for 20 h. After the culture, the whole blood samples at 1000 × RCF (g) for 5 min were centrifuged. Then, the ID card was removed, confirming the batch number is consistent with the test card, and the ID card inserted into the chip port. Eighty microlitres of samples was sucked and added into the hole of test card. The instrument will automatically test and output the results within 15 min (Figure 1). The levels of IFN-γ (pg/mL) in N, T, and P were detected with the results being expressed as T-N and P-N, respectively. The definition of positive was that the IFN-γ values of T-N were ≥ 20 pg./mL and ≥ 25% of the N value, while the negative result was that the IFN-γ values of T-N were ≥20 pg/mL and (T-N < 25% of the N value and P-N ≥ 25 pg/mL), or the IFN-γ values of T-N were <20 pg/mL and P-N ≥ 25 pg/mL. The definition of indeterminate was that the IFN-γ value of the N tube was greater than 1000 pg/mL, or the IFN-γ value of the N tube was ≤1000 pg/mL and (T-N ≥ 20 pg/mL, T-N < 25% N and P-N < 25 pg/mL), or the IFN-γ value of the N tube was ≤1000 pg/mL and (T-N < 20 pg/mL and P-N < 25 pg/mL).

### Statistical analysis

Data entry was completed by EpiData software (EpiData Version 3.1, EpiData Association Odense, Denmark). Data analysis was conducted by Statistical Analysis System (SAS 9.4; SAS Institute Inc., NC, USA) and GraphPad Prism 8 (GraphPad Software, San Diego, CA).

Data are described as number (percentage) or median value and interquartile range (IQR). Income per month was classified by the local average level in 2021 (2500 RMB [Ren Min Bi]) [8]. Body mass index (BMI) was divided into four categories: underweight (< 18.5 kg/m^2^), normal weight (18.5–24.0 kg/m^2^), overweight (24.0–28.0 kg/m^2^), or obese (≥ 28.0 kg/m^2^). The indeterminate results of the two tests were reported but not included in analyses.

Pearson’s Chi-squared test compared the categorical variables across groups. Univariate analysis would include variables that showed significant relations in multivariate analysis (logistic regression analyses). Cohen’s kappa coefficient (κ) was used to evaluate the consistency between the two tests. κ >0.75 is usually considered good consistency, and κ < 0.4 means poor agreement. The IFN-γ levels between different groups were compared by the Wilcoxon rank sum test. Classify variables based on negative consistency (both tests are negative) or positive consistency (both tests are positive) to identify risk factors associated with MTB infection. The significance level was 0.05 in this study.

## Result

### Characteristics of study participants

Of 625 registered village doctors, the examination was performed in 519 (83.04%). After excluding 8 individuals with incomplete data, 511 individuals were included in the final statistical analysis.

The major characteristics of the participants are shown in Table 1. Overall, 62.62% (320/511) of the participants were males, and 81.02% (414/511) of the participants were older than 40 years. About a quarter (23.29%, 119/511) of the participants ever smoked and 25.05% (128/511) reported current alcohol drinking. About two-thirds of the village doctors were overweight or obese. 52.64% (269/511) of the participants had ≥ 26 years of service as rural doctors and a quarter of participants had ever managed patients with TB in the past 4 years. The positivity rate of the QFT-Plus test and the AIMTB rapid test assay was 20.94% (107/511) and 24.85% (127/511), respectively. Indeterminate results were reported for 3 samples in each of the two tests.

**Table 1.** Demographics of the study population

	n	%
**Total**	**511**	**100**
**Age (years)**		
20–29	14	2.74
30–39	83	16.24
40–49	132	25.83
50–59	185	36.20
≥60	97	18.98
**Sex**		
Female	191	37.38
Male	320	62.62
**Income per month** ^a^		
<2500 RMB	180	35.23
≥2500 RMB	331	64.77
**Education level**		
Middle school or lower	14	2.74
High school	340	66.54
College or higher	157	30.72
**Alcohol drinking (within past 12 months)**		
No	383	74.95
Yes	128	25.05
**Smoking**		
Never smoked	392	76.71
Ever smoked	119	23.29
**BMI (kg/m**^**2**^**)**		
< 18.5	9	1.76
18.5–24.0	175	34.25
24.0–28.0	203	39.73
≥ 28.0	124	24.27
**Years of service as a rural doctor** ^b^		
< 26	242	47.36
≥ 26	269	52.64
**History of TB**		
No	509	99.61
Yes	2	0.39
**Ever managed patients with TB in the past 4 years**		
No	384	75.15
Yes	127	24.85
**QFT-Plus test**		
Positive	107	20.94
Negative	401	78.47
Indeterminate	3	0.59
**AIMTB rapid test assay**		
Positive	127	24.85
Negative	381	74.56
Indeterminate	3	0.59

Abbreviation: BMI, body mass index; TB, tuberculosis; QFT-Plus, QuantiFERON-TB Gold Plus.

a Stratified by the local average level.

b Stratified by the value of median.

### Risk factors for MTB infection

The results of risk factors related to MTB infection are shown in Table 2. Being male (odds ratio [OR] = 2.33, 95% confidence interval [CI]: 1.23–4.43) and with ≥26 years of service as rural doctors (OR = 2.44, 95% CI: 1.26–4.72) were significantly associated with MTB infection.

**Table 2.** Factors associated with MTB infection among village doctors

	MTB infection^a^, n/N (%)	p for Pearson’s Chi-squared test	Adjusted OR (95% CI)^b^
**Age (years)**		**<0.001**	
<50	29/217 (13.36)		Ref
≥ 50	74/261 (28.35)		1.13 (0.59, 2.17)
**Sex**		**<0.001**	
Female	17/178 (9.55)		Ref
Male	86/300 (28.67)		**2.33 (1.23, 4.43)**
**Income per month** ^c^		0.997	
<2,500 RMB	36/167 (21.56)		
≥ 2,500 RMB	67/311 (21.54)		
**Smoking**		**0.004**	
Never smoked	67/363 (18.46)		Ref
Ever smoked	36/115 (31.30)		1.25 (0.74, 2.11)
**Education level**		**0.029**	
High school or lower	80/329 (24.32)		Ref
College or higher	23/149 (15.44)		1.00 (0.56, 1.78)
**Alcohol drinking (within past 12 months)**		0.304	
No	72/353 (20.40)		
Yes	31/125 (24.80)		
**BMI (kg/m**^**2**^**)**		**0.041**	
< 18.5	1/9 (11.11)		0.83 (0.09, 7.69)
18.5–24.0	25/164 (15.24)		Ref
24.0–28.0	52/190 (27.37)		1.44 (0.81, 2.55)
≥ 28.0	25/115 (21.74)		0.96 (0.56, 1.78)
**Years of service as rural doctors** ^d^		**<0.001**	
<26	26/229 (11.35)		Ref
≥26	77/249 (30.92)		**2.44 (1.26, 4.72)**
**Ever managed patients with TB in the past 4 years**		0.471	
No	75/361 (20.78)		
Yes	28/117 (23.93)		

Abbreviation: MTB, *Mycobacterium tuberculosis*; BMI, body mass index; TB, tuberculosis.

a Classify variables based on negative consistency (both tests are negative) or positive consistency (both tests are positive).

b Adjusted for age, sex, smoking, years of service, education level, and BMI.

c Stratified by the local average level.

d Stratified by the value of median.

### Agreement analysis of the AIMTB rapid test assay and the QFT-Plus test

As shown in Table 3, five participants with indeterminate results of the two tests were excluded from the agreement analysis. The remaining 506 results showed that the concordance between the two tests was 94.47% (κ = 0.84, 95% CI: 0.79–0.90).

**Table 3.** Concordance between the QFT-Plus test and the AIMTB rapid test assay

	QFT-Plus − / AIMTB − n^a^ (%)	QFT-Plus + / AIMTB + n^a^ (%)	QFT-Plus + / AIMTB − n^a^ (%)	QFT-Plus − / AIMTB + n^a^ (%)	Kappa value (95% CI)	Concordant (%)
**Total (N = 506)**	375 (74.11)	103 (20.36)	4 (0.79)	24 (4.74)	0.84 (0.79, 0.90)	94.47
**Stratified by sex**						
Female (N = 187)	161 (86.10)	17 (9.09)	1(0.53)	8 (4.28)	0.76 (0.62,0.91)	95.19
Male (N = 319)	214 (67.08)	86 (26.96)	3(0.94)	16 (5.02)	0.86 (0.80, 0.92)	94.04
**Stratified by years of service**						
Years of service <26 (N = 239)	203 (84.94)	26 (10.88)	1 (0.42)	9 (3.77)	0.82 (0.70, 0.93)	95.82
Years of service ≥26 (N = 267)	172 (64.42)	77 (28.84)	3 (1.12)	15 (5.62)	0.85 (0.78, 0.91)	93.26

Abbreviation: QFT-Plus, QuantiFERON-TB Gold Plus; AIMTB, AIMTB rapid test assay.

a Indeterminate results of the QFT-Plus test and the AIMTB rapid test assay were excluded.

Agreement between the two tests was higher in males (κ = 0.86, 95% CI: 0.80–0.92) than in females (κ = 0.76, 95% CI: 0.62–0.91). In addition, the agreement was found to be higher in participants with ≥26 years of service (κ = 0.85; 95% CI: 0.78–0.91) than in those with less service (κ = 0.82; 95% CI: 0.70–0.93) (Table 3).

### IFN-γ releasing levels among participants with concordant and discordant results

**Figure 2.**
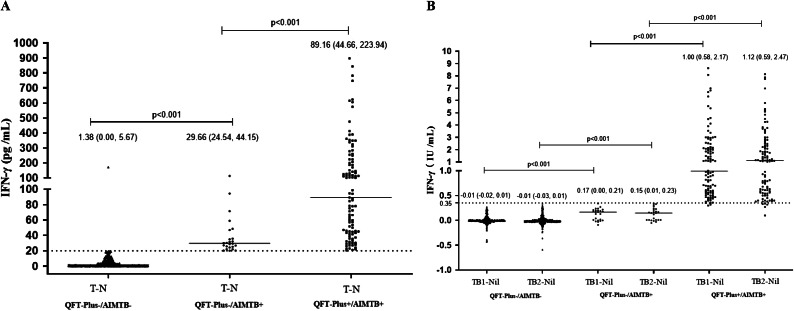
IFN-γ releasing levels between concordant and discordant results of the AIMTB rapid test assay and the QFT-Plus test. (a) Quantitative IFN-γ responses of the AIMTB rapid test assay were compared between QFT-Plus-/AIMTB**−**, QFT-Plus+/AIMTB+ and QFT-Plus-/AIMTB+. (b) Quantitative results of the QFT-Plus were compared between QFT-Plus-/AIMTB**−**, QFT-Plus+/AIMTB+ and QFT-Plus-/AIMTB+. Horizontal lines represent median IFN-γ levels. Data were presented as median concentration and interquartile range (IQR). The differences were tested by the Wilcoxon rank sum test.

The participants were classified into the following groups: QFT-Plus-/AIMTB-, QFT-Plus+/AIMTB+, QFT-Plus-/AIMTB+, and QFT-Plus+/AIMTB-. The median value of IFN-γ in the AIMTB rapid test assay among participants of QFT-Plus-/AIMTB+ was 29.66 (IQR: 24.54–44.15) pg./mL, which was dramatically higher than among participants of QFT-Plus-/AIMTB- with a median value of 1.38 (IQR: 0.00–5.67) pg./mL (p < 0.001). Additionally, it was significantly lower than among participants of QFT-Plus+/AIMTB+ with a median value of 89.16 (IQR: 44.66–223.94) pg./mL (p < 0.001) (Figure 2). For the QFT-Plus test, the level of IFN-γ among participants of QFT-Plus-/AIMTB+ was found to be higher than among participants of QFT-Plus-/AIMTB- as well.

## Discussion

As we know, our study is the first to use QFT-Plus test as reference to assess the performance of AIMTB rapid test assay in a Chinese population. The consistency rate of the two tests was 94.47% (κ = 0.84), which was observed to be improved in subgroups with higher risk of MTB infection. The results support the application of the AIMTB rapid test assay for LTBI testing at the community level to promote TB preventive treatment in high-risk populations.

In the present study, the AIMTB rapid test assay showed good agreement with the QFT-Plus test. This was consistent with the findings reported for another IGRA assay based on immunofluorescence technology (QIAreach) [9], which showed excellent concordance with QFT-Plus test (κ = 0.88) in close contact with active patients with TB. Besides, if the two detection methods show similar sensitivity, the consistency between the two detection methods might improve as the prevalence of TB increases in the target population [10,11]. Consistently, in this study, we found that the agreement was improved in subgroups at higher risk of infection, such as in the participants with ≥26 years of service. Additionally, the quantitative results of the QFT-Plus test in our study were significantly higher in participants of QFT-Plus-/AIMTB+ than in those of QFT-Plus-/AIMTB-. It can thus be proposed that the AIMTB rapid test assay might have higher positive prediction value. Although the AIMTB rapid test assay performed reasonably well as compared with the widely applied method, independent evaluations of the sensitivity and the specificity are necessary based on the reference gold standard.

In addition, the LTBI prevalence among village doctors in our study was 20.94% based on the QFT-Plus test, which was lower than that previously reported in 2017 (31.22%) (*p* < 0.05) [12]. A possible explanation for such a difference might be that the proportion of males in this study was lower than in 2017 (62.62% vs. 68.34%, *p* < 0.05), as men were at higher risk of MTB infection than women [12]. Another possible explanation might be that the village doctors’ awareness of self-protection has increased after the experience of COVID-19. For example, in our study, we found that approximately 90% of the village doctors wore masks when they engaged in TB diagnosis and treatment. However, in the survey conducted in 2017, the proportion was only 32%. Besides, our study also found that participants with ≥26 years of service were more likely to be infected with MTB. This observation was consistent with a number of previous studies, that is, the years of service as healthcare workers in TB-related sectors was an important risk factor for MTB infection [13,14]. It suggests the urgent need to improve MTB infection control in such an occupational-exposure population.

Our study had several limitations. First, due to the limited sample size, we could not identify potential factors associated with discordant results. Second, we used a manufacture suggested cutoff value of 20 pg./mL for the AIMTB rapid test assay, which has not been widely validated in Chinese populations. Therefore, our results could not represent the results in other populations or study settings. Third, sociodemographic data and information on potential risk factors were obtained through the face-to-face questionnaires. Information bias due to incorrect answers could not be ruled out. Last, as the performance of MTB infection testing might be influenced by the actual infection rate in various populations, validation and evaluation of this new tool in different populations are needed in the future to optimize its application.

## Conclusion

In conclusion, the AIMTB rapid test assay was found to be a reliable novel tool for MTB infection testing. As it does not require complex detection techniques, reports result faster and at a lower cost, it is more suitable for serving LTBI testing and treatment in key populations at a community level, especially in resource-limited settings. Our findings need validation along with the application of this new method in more populations.

## Data Availability

The data of this study are available upon reasonable request.
